# Sendai virus, an RNA virus with no risk of genomic integration, delivers CRISPR/Cas9 for efficient gene editing

**DOI:** 10.1038/mtm.2016.57

**Published:** 2016-08-24

**Authors:** Arnold Park, Patrick Hong, Sohui T Won, Patricia A Thibault, Frederic Vigant, Kasopefoluwa Y Oguntuyo, Justin D Taft, Benhur Lee

**Affiliations:** 1 Department of Microbiology, Icahn School of Medicine at Mount Sinai, New York, New York, USA

## Abstract

The advent of RNA-guided endonuclease (RGEN)-mediated gene editing, specifically via CRISPR/Cas9, has spurred intensive efforts to improve the efficiency of both RGEN delivery and targeted mutagenesis. The major viral vectors in use for delivery of Cas9 and its associated guide RNA, lentiviral and adeno-associated viral systems, have the potential for undesired random integration into the host genome. Here, we repurpose Sendai virus, an RNA virus with no viral DNA phase and that replicates solely in the cytoplasm, as a delivery system for efficient Cas9-mediated gene editing. The high efficiency of Sendai virus infection resulted in high rates of on-target mutagenesis in cell lines (75–98% at various endogenous and transgenic loci) and primary human monocytes (88% at the *ccr5* locus) in the absence of any selection. In conjunction with extensive former work on Sendai virus as a promising gene therapy vector that can infect a wide range of cell types including hematopoietic stem cells, this proof-of-concept study opens the door to using Sendai virus as well as other related paramyxoviruses as versatile and efficient tools for gene editing.

## Introduction

The explosion of research into CRISPR/Cas9-mediated gene editing is driven by its clear advantages in ease of use and efficiency over previous methods. Upon targeting of Cas9 to a specific locus on dsDNA, Cas9 cleaves both DNA strands to create a double-strand break, often inducing mutagenesis via nonhomologous end joining or promoting homologous recombination in the presence of a donor template.^1–3^ In contrast to TALEN- and ZFN-based methods, which rely on careful and often arduous optimization of protein-based parameters for each DNA target, targeting of Cas9 to specific genomic loci is determined by simple Watson-Crick base pair matching between a short (~20 bp) portion of the Cas9-associated guide RNA and the dsDNA target sequence.^4,5^ The only restraint on target selection is the requirement for a short PAM (protospacer adjacent motif) sequence, specific for each Cas9 variant, to be present in the target DNA following the matching target sequence. Since the initial discovery of the mechanism and function of CRISPR/Cas9 (refs. 1,2) and the demonstration that this bacteria-derived system could be successfully applied in mammalian cells,^6–8^ many efforts and advances have been made to improve CRISPR/Cas9 delivery, efficiency, and specificity.^3–5,9^

Although nonviral delivery systems show promise,^10,11^ viral delivery systems have critical advantages that make them the method of choice for most gene-editing applications.^12,13^ Viruses have evolved to target specific cell types, efficiently deliver genetic information, and recruit cellular factors to aid expression of virus-encoded genes. The viral systems used today for delivery of CRISPR/Cas9, lentivirus and adeno-associated virus (AAV), have successfully modified cells both *ex vivo* and *in vivo*. Although DNA-based replication of these viruses carries the risk of unwanted integration into the host genome and potential genotoxicity or oncogenesis,^14,15^ the latest generation of these vectors have minimized these risks to a large extent. Nonetheless, despite much attention to this problem and innovations such as the use of integration-defective lentivirus, undesirable integration remains a carefully monitored risk that may affect the success of future gene therapy trials.^16–19^

To complement existing DNA-based viral delivery systems, we turned to an RNA virus with no DNA intermediate and no nuclear phase in its lifecycle, thus eliminating the risk of unwanted integration.^20–22^ Sendai virus (SeV), long a scourge of laboratory mouse colonies, is a paramyxovirus (order *Mononegavirales*, family *Paramyxoviridae*, genus *Respirovirus*) closely related to human parainfluenza virus-1 and -3. Among its advantages is safety, as despite having been worked with extensively in laboratories for decades, SeV has never been linked to human disease.^22,23^ SeV further has a broad cellular tropism, using ubiquitous sialic acid as the cellular receptor, and readily infects many tissue and cell types including airway epithelium,^24^ hematopoietic stem cells,^25,26^ monocytes, macrophages, and dendritic cells,^27,28^ and endothelial, muscle, and neuronal cells^29^; it replicates to high titers in cell culture and in chicken eggs^22^; and it can readily accommodate and robustly express foreign genes.^30^ These advantages led to its ongoing development as a gene therapy vector in clinical trials,^31^ as well as its current use as a commercial vector for induction of pluripotency to generate pluripotent stem cells^26,32^ (Invitrogen).

We therefore inserted both the *S. pyogenes* Cas9 as well as its associated guide RNA as separate transcriptional units within the SeV genome. A major challenge we faced was that guide RNA function depends on a precise start and end to the guide RNA sequence, which is usually provided on a DNA template by the use of a nuclear Pol III promoter such as U6 to drive RNA expression, in combination with a terminator sequence immediately following the guide RNA.^3,6^ To accomplish this in the context of a purely RNA-dependent and cytoplasmic replication lifecycle, we inserted hammerhead ribozymes immediately preceding and following the guide RNA. Upon expression of the “mRNA” transcript encoding the guide RNA from the viral genome, the flanking ribozymes would self-cleave and precisely liberate the guide RNA. Using reverse genetics, we rescued replication-competent SeV encoding Cas9 and its associated guide RNA. This recombinant SeV-Cas9 virus achieved almost complete (98%) mutagenesis of a reporter gene in the cognate reporter cell line, as well as high rates of mutagenesis (~75–90%) of endogenous alleles in HEK293s and primary human monocytes without any need for selection for transduced cells. These findings open the door to development of Sendai virus and related paramyxoviruses as vectors for efficient delivery of CRISPR/Cas9 without the risk of undesirable integration into host genomes.

## Results

### Sendai virus incorporating Cas9 and a guide RNA flanked by self-cleaving ribozymes replicates to high titer

**Figure 1:**
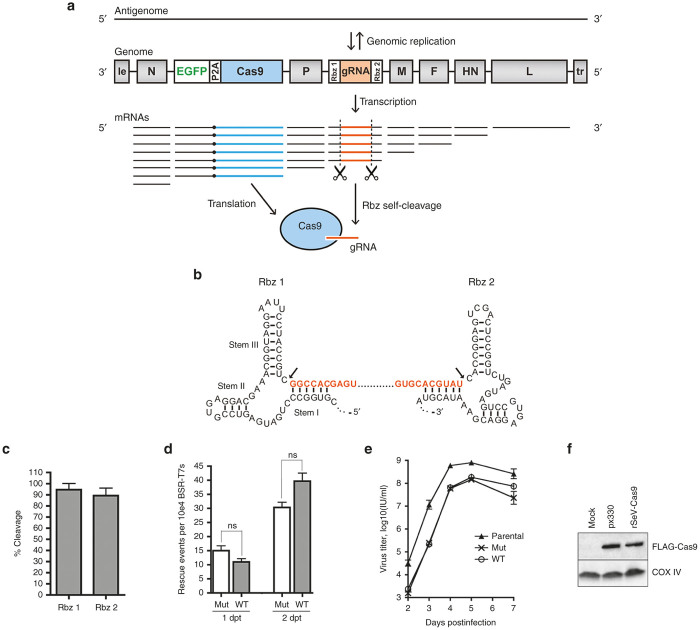
Sendai virus incorporating Cas9 and a guide RNA flanked by self-cleaving ribozymes replicates to high titer. (**a**) The negative-sense RNA genome is flanked by virus promoters (the 3’ leader (le), which serves as the genomic promoter, and the 5’ trailer (tr), which serves as the antigenomic promoter). Shown are the Sendai virus genes N (nucleoprotein), P (phosphoprotein), M (matrix), F (fusion protein), HN (attachment protein), and L (large RNA-dependent RNA polymerase). An EGFP-P2A-Cas9 cassette (5.1 kb) was inserted between N and P, and a guide RNA flanked by self-cleaving ribozymes (rbz 1 and 2) (0.2 kb total) was inserted between P and M (see Materials and Methods for further details). The ribozymes are only functional in the positive-sense, or 5’-to-3’, orientation. Genome may be transcribed from 3’ to 5’ into either full length antigenome or individual capped and polyadenylated mRNAs. These mRNAs are produced in a polar transcriptional gradient, with N mRNAs being the most abundant, and L mRNAs being the least abundant. (**b**) The self-cleaving hammerhead ribozyme sequences and structures are shown. The chimeric guide RNA is shown in orange, corresponding to the orange highlight in panel **a**. Arrows indicate sites of cleavage. (**c**) The self-cleavage activity of the ribozymes was assayed by qRT-PCR as described in Materials and Methods. Error bars represent standard deviation from 3 independent experiments. (**d**) rSeV-Cas9 (WT), or rSeV-Cas9 with both ribozymes mutated to abolish self-cleavage (Mut) (see Supplementary Figure S1 for mutations), was rescued from plasmid DNA as described in Materials and Methods. As EGFP is only expressed upon conversion of transfected antigenome to genome and subsequent virus mRNA production, rescue efficiency was determined by observing GFP+ cells (rescue events) by flow cytometry at 1–2 days post-transfection (dpt). Error bars represent standard deviation from 3 replicates. ns, not significant. (**e**) BSR-T7 cells were infected at a multiplicity of infection (MOI) of 0.01. The parental SeV has the EGFP reporter but lacks Cas9 and the guide RNA cassette. Error bars represent standard deviation from three replicates. There was no significant difference (*P* > 0.05) between WT and Mut at any time point, two-way ANOVA followed by Bonferroni post-tests. (**f**) HEK293 cells in six-well were transfected with 2 ug px330 (from which the FLAG-tagged Cas9 in rSeV-Cas9 was derived)^6^ or infected with rSeV-Cas9 at a MOI of 10. Cell lysates were collected 2 days later and processed via SDS-PAGE and Western blot analysis for detection of the FLAG epitope on Cas9. COX IV represents the loading control.

Paramyxoviruses have a single-stranded, negative-sense RNA genome. During replication, the virus replication complex (nucleoprotein (N), phosphoprotein (P), and large RNA-dependent RNA polymerase (L)) uses the genome as a template for production of both full length antigenome (the reverse complement of the genome) and individual capped and polyadenylated mRNAs (Figure 1a). The antigenome is further transcribed into genome, thus amplifying the genome for replication. During mRNA production, gene start and gene stop signals within the flanking intergenic regions determine the ends of the mRNA transcript (see Supplementary Figure S1 for an example). For this proof-of-principle study, we used our recombinant SeV (rSeV) with enhanced green fluorescent protein (EGFP) inserted between the N and P genes via duplication of the N-to-P intergenic region.^33,34^ We inserted *S. pyogenes* Cas9 downstream of the EGFP reporter via a P2A ribosomal skipping sequence (Figure 1a). We further inserted a chimeric guide RNA (20 bp target sequence and 76 bp trans-activating CRISPR RNA) as a new “gene” between the P and M genes via duplication of the P-to-M intergenic region (Figure 1a and Supplementary Figure S1). The guide RNA was flanked by self-cleaving hammerhead ribozymes to provide precise ends to the guide RNA (Figure 1a,b and Supplementary Figure S1).

We first confirmed that the ribozymes were functional for cleavage by transfecting the DNA construct encoding the T7-driven rSeV-Cas9 positive-sense antigenome (the ribozymes are functional in the RNA positive-sense orientation) into BSR-T7 cells (BHK cells stably expressing T7 polymerase). Quantitative reverse transcription PCR (qRT-PCR) on T7-transcribed antigenomic RNA extracted from transfected cells showed efficient self-cleavage for both ribozymes (Figure 1c). We then attempted to rescue replication-competent rSeV-Cas9 by co-transfecting the antigenome construct with the accessory SeV-N, -P, and -L expression constructs required for genomic replication and thus virus rescue. We initially supposed that rescue efficiency and/or genomic replication might be impaired or even blocked by the presence of self-cleaving ribozymes in the antigenome. However, we hypothesized that nucleoprotein encapsidation of the antigenomic RNA would happen quickly enough to prevent formation of the ribozyme structure and thus self-cleavage of the antigenome; by contrast, mRNAs are not encapsidated, and thus the mRNA encoding the guide RNA would be free to undergo ribozyme cleavage. To our surprise, we found that rSeV-Cas9 (WT) rescued as efficiently as a corresponding control virus (Mut) with mutations in the ribozymes to prevent ribozyme activity (Figure 1d). Further, the growth kinetics of rSeV-Cas9 matched those of the control virus, consistent with the lack of a negative effect of the ribozymes on genomic replication (Figure 1e). As expected from the addition of almost 5 kb of additional sequence to the genome, both Cas9-modified viruses peaked at ~0.5 log lower titers than the parental SeV without Cas9 or the guide RNA cassette (Figure 1e), although they still reached peak titers of ~10^8^ IU/ml, consistent with standard peak titers for SeV in cell culture.^22^

Finally, we confirmed that rSeV-Cas9 produced the Cas9 protein upon infection. Western blot analysis of HEK293 cells either transfected with a Cas9-expressing plasmid or infected with rSeV-Cas9 showed the expression of Cas9 protein (Figure 1f).

### rSeV-Cas9 targeting mCherry gene achieves almost complete mutagenesis of a reporter cell line

**Figure 2:**
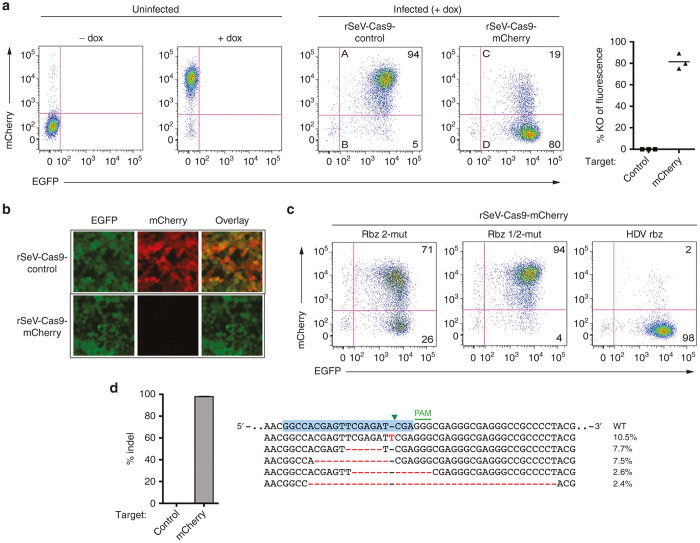
rSeV-Cas9 targeting mCherry gene achieves almost complete mutagenesis of a reporter cell line. (**a**) mCherry-inducible HEK293 cells were infected with rSeV-Cas9-control (no guide RNA) or rSeV-Cas9-mCherry (guide RNA targeting mCherry) at multiplicity of infection (MOI) 25. Expression of mCherry was induced with doxycycline (dox) after 4 days postinfection, and cells were collected for flow cytometry the following day. Percent knockout (KO) of mCherry fluorescence was determined as 100*(1−(C/(C + D))/(A/(A + B))). Results from three independent experiments are shown. (**b**) Cells treated as in panel **a** were imaged by fluorescence microscopy. The same exposure was used for each condition. (**c**) rSeV-Cas9-mCherry was mutated to render Rbz 2 (Rbz 2-mut) or both ribozymes (Rbz 1/2-mut) nonfunctional. An alternative 3’ ribozyme, the hepatitis delta virus (HDV) ribozyme (see Supplementary Figure S1 for sequence), was also tested via replacement of Rbz 2. The experiment was performed as in panel **a**. (**d**) HEK293 cells were infected with rSeV-Cas9-control or rSeV-Cas9-mCherry at MOI 25 and collected for deep sequencing of the mCherry locus at 6 days postinfection. Error bars represent Jeffreys 95% confidence intervals. The five most abundant species of mutated target and their relative abundance percentages are shown. Blue highlight represents the 20 bp target sequence, green arrowhead represents the Cas9 cleavage site, and the 3 bp PAM motif is shown. Mutations are in red font.

Our initial rSeV-Cas9 incorporated a guide RNA specific for the mCherry gene (rSeV-Cas9-mCherry). We created a HEK293-based reporter cell line with inducible mCherry, and we infected this cell line at a multiplicity of infection (MOI) of 25 with either rSeV-Cas9-mCherry or a control virus expressing Cas9 but lacking the guide RNA cassette (rSeV-Cas9-control). Induction of mCherry expression at various days postinfection showed a progression of knockout over time, with knockout appearing more pronounced starting at 4 days postinfection (Supplementary Figure S2 and Figure 2a). Quantification of this time point (induction at day 4 and collection for flow cytometry at day 5) showed ~80% knockout of mCherry fluorescence (Figure 2a). Fluorescence microscopy visually confirmed the strong reduction of mCherry fluorescence upon knockout (Figure 2b).

We also used the reporter cell line to confirm the requirement for the ribozymes to preserve guide RNA function. Mutation of the 3’ ribozyme (rbz 2) strongly reduced reporter knockout efficiency, while mutation of both the 5’ and 3’ ribozymes (rbz 1/2) abrogated knockout activity (Figure 2c, compare to Figure 2a). This result underlines the importance of the ribozymes and the precise RNA ends that they generate. We further tested an alternative 3’ ribozyme, the widely-used hepatitis delta virus ribozyme, in place of the existing hammerhead ribozyme. This version of rSeV-Cas9-mCherry also efficiently knocked out mCherry fluorescence, perhaps with even greater efficiency (Figure 2c).

Some nonframeshift mutations might not result in knockout of mCherry fluorescence. To quantitatively assess the degree of mutagenesis induced by rSeV-Cas9-mCherry, we performed deep sequencing on the mCherry locus amplified from reporter cells collected at day 6 postinfection. We found that 98% of alleles had indels, indicating nearly complete mutagenesis of the reporter (Figure 2d). These results suggested that the rSeV-Cas9 vector might prove to be highly efficient in targeting endogenous alleles as well.

### rSeV-Cas9 efficiently mutates endogenous *ccr5* and *efnb2*

**Figure 3:**
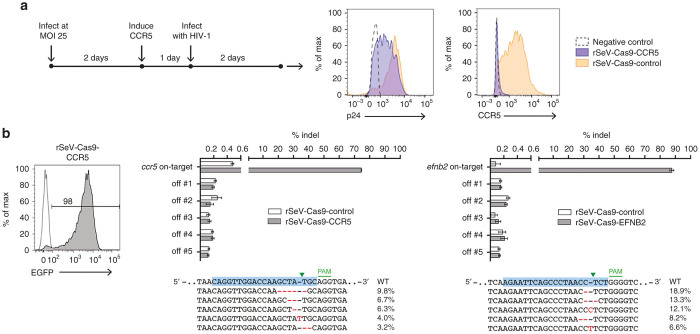
rSeV-Cas9 efficiently mutates endogenous *ccr5* and *efnb2*. (**a**) Affinofile cells^35^ were infected with rSeV-Cas9-control or rSeV-Cas9-CCR5 at multiplicity of infection (MOI) 25. CD4/CCR5 overexpression was induced at day 2, and cells were further infected with CCR5-tropic HIV-1 the following day. Flow cytometry for p24 and CCR5 was performed 5 days after infection with rSeV. Data shown is gated on rSeV-infected cells (GFP+). For p24, the negative control is stained cells uninfected with HIV. For CCR5, the negative control is unstained cells. (**b**) HEK293 cells were infected with rSeV-Cas9-control or the targeting viruses rSeV-Cas9-CCR5 or rSeV-Cas9-EFNB2 at MOI 25. Flow cytometry at 2 days postinfection indicated 98% infection. Cells were collected at 6 days postinfection for deep sequencing of target and off-target loci (see Supplementary Figure S5 for genomic locations and sequences). Error bars represent Jeffreys 95% confidence intervals. For each target, the five most abundant species of mutated target and their relative abundance percentages are shown.

As opposed to the single allele of mCherry in our reporter cell line, there are two or more alleles of most endogenous genes per cell. To test the ability of our Sendai virus vector to target the more abundant endogenous alleles, we generated rSeV-Cas9 viruses targeting coding exons of the human *ccr5* and *efnb2* genes. We first performed a preliminary test of the ability of rSeV-Cas9-CCR5 to induce mutagenesis resulting in functional disruption of *ccr5*. Since HEK293 cells express negligible levels of CCR5, we used HEK293-based Affinofile cells, which contain inducible CD4 and CCR5 transgenes in addition to their endogenous alleles.^35^ CD4 and CCR5 are cell surface receptors required for infection by R5-tropic HIV-1, and Affinofile cells have been used extensively to characterize CCR5-mediated HIV entry.^35–37^ We infected Affinofile cells with rSeV-Cas9-CCR5, and at 2 days postinfection, CD4/CCR5 overexpression was induced, and the cells were further infected with an R5-tropic HIV-1 isolate the following day (Figure 3a). At this early time point, we expected cells infected with rSeV-Cas9-CCR5 to have lower levels of CCR5 relative to cells infected with rSeV-Cas9-control due to ongoing mutagenesis of the inducible *ccr5* transgene and endogenous *ccr5* alleles. After an additional 2 days, flow cytometry revealed efficient knockout of the induced CCR5 by this final time point. p24 staining indicative of HIV-1 infection at the earlier time point had a 51% reduction in geometric mean fluorescence intensity compared to the rSeV-Cas9-control infection (Figure 3a), with remaining infection likely due to incomplete mutagenesis by the earlier time point.

To examine mutagenesis of endogenous alleles, we infected HEK293 cells with the *ccr5*- and *efnb2*-targeting rSeV-Cas9 viruses at a MOI of 25, collected the cells at 6 days postinfection, and PCR-amplified the on-target loci as well as the top five predicted off-target sites. We note that HEK293 cells are known to generally have 3 copies of chromosome 3 (encoding *ccr5*) and two to three copies of chromosome 13 (encoding *efnb2*).^38^ Deep sequencing revealed high rates of on-target mutagenesis (75 and 88% for *ccr5* and *efnb2*, respectively) (Figure 3b). Off-target mutagenesis was unremarkable for this first-generation Cas9 without modifications to increase specificity, ranging from no detectable increase to 0.05% above the nontargeting control (Figure 3b, observe relative indel frequencies as compared to the control). These results confirmed that Sendai virus delivery of CRISPR/Cas9 can efficiently target endogenous genes.

### *Ccr5*-targeting rSeV-Cas9 edits primary human monocytes at high frequency

**Figure 4:**
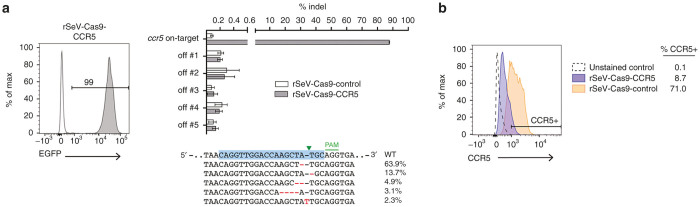
*Ccr5*-targeting rSeV-Cas9 edits primary human monocytes at high frequency. (**a**) Primary human monocytes were infected with rSeV-Cas9-control or rSeV-Cas9-CCR5 at multiplicity of infection (MOI) 50 with simultaneous stimulation with GM-CSF and collected at 5 days postinfection for deep sequencing of on-target and off-target loci. Flow cytometry showed 99% infection. Error bars represent Jeffreys 95% confidence intervals. For each target, the five most abundant species of mutated target and their relative abundance percentages are shown. (**b**) Primary human monocytes from an independent donor were infected as in panel **a**, and cells were collected at 5 days postinfection for flow cytometry of cell surface CCR5. Data shown is gated on infected cells (GFP+).

Finally, we confirmed that rSeV-Cas9 can efficiently mutate freshly isolated primary cells. We infected primary human CD14+ monocytes, which are normally resistant to lentiviral transduction, with rSeV-Cas9-CCR5 at a MOI of 50. To better visualize reduction in CCR5 expression upon mutagenesis, monocytes were also stimulated with GM-CSF to induce macrophage differentiation with concomitant upregulation of CCR5. Cells were collected at 5 days postinfection, and deep sequencing revealed 88% on-target mutagenesis (Figure 4a). It was interesting to note that the two single-nucleotide deletions flanking the cleavage site together comprised 78% of all detected indels (Figure 4a and Supplementary Figure S3); by contrast, in HEK293 cells, the same deletions together comprised 9% of detected indels, and no single mutation comprised more than 10% of the total (Figure 3b and Supplementary Figure S3). Infection of monocytes from an independent donor showed a similar result, with the above deletions comprising ~50% of mutant alleles (19/38 mutations via Sanger sequencing), indicating that this may represent a cell type-specific phenomenon. When single specific mutations comprise such a large proportion of the total indels, mismatch-based assays such as the T7E1 endonuclease assay, which relies on highly variable mutagenesis to detect mutations, may strongly underestimate the degree of on-target mutagenesis.^39^ As with the HEK293 cells, detected mutagenesis of predicted off-target loci in the monocytes was negligible (Figure 4a). Flow cytometry of infected monocytes from an independent donor confirmed knockout of cell surface CCR5 at the same time point (Figure 4b).

## Discussion

In this work, we show that Sendai virus, an RNA virus with no DNA or nuclear phase in its lifecycle, can be repurposed to deliver CRISPR/Cas9 to cells for efficient gene editing. To do so, we overcame the critical barrier of incorporating guide RNAs into the SeV genome by flanking the guide RNA with self-cleaving ribozymes (Figure 1a,b). With this result, we have found for the first time that SeV, and thus likely other paramyxoviruses, can tolerate self-cleaving ribozymes within the genome, likely due to cotranscriptional encapsidation of the genomic and antigenomic RNA by the nucleoprotein and thus prevention of ribozyme activity during replication of the full-length RNA. Along with further incorporation of Cas9 expression, the rescued replication-competent virus was able to efficiently induce mutagenesis of the guide RNA target sequence in the genome. For example, although the efficiency of our *ccr5*-targeting virus is not directly comparable to other studies due to the differing guide RNA sequences and target cells used, we achieved rates of *ccr5* mutagenesis (75–88%) similar to or higher than those achieved via lentivirus or AAV CRISPR/Cas9 transduction.^40–42^ Further, because SeV infection was highly efficient, achieving these high rates of mutagenesis did not require sorting or selection for infected cells.

In addition to the advantages of broad tropism, growth to high titers, and robust expression of foreign genes previously mentioned, SeV has additional important advantages as a gene therapy vector. First, paramyxoviruses are amenable to envelope switching or modification, in which envelope proteins with different cell type specificities can be substituted for the original, or the original attachment or fusion protein itself can be modified to have a different specificity.^43–45^ Second, SeV, like other paramyxoviruses, has a polar transcriptional gradient (Figure 1a) with reduction of transcript levels as the polymerase complex moves from the 3’ to 5’ end of the genome.^20^ The efficiency versus the specificity of Cas9 activity appears to be a trade-off,^2,3,46,47^ and the optimal levels of Cas9 and guide RNA expression therefore likely must be determined for each CRISPR delivery platform. Thus, for paramyxoviruses, levels of Cas9 and guide RNA expression can be modulated and fine-tuned by shifting the insertion position of these introduced elements within the genome, or by modifying the strength of gene start signals.^48,49^ Third, paramyxoviruses are not prone to genetic recombination or instability, and no homologous or heterologous recombination has ever been detected for SeV.^22^ Fourth, despite a high prevalence of immunity to the related human parainfluenza virus-1, cross-neutralizing anti-SeV titers are low.^50^ Thus, SeV, as a mouse pathogen, would not encounter significant pre-existing specific immunity in humans.

In this proof-of-principle study, we used a typically cytopathic wild-type strain of SeV, thus limiting functional studies that can be performed with edited cells postinfection. Importantly, SeV has been extensively studied and modified to develop temperature-sensitive, noncytopathic, and replication-incompetent Sendai viruses that are useful for *ex vivo* and *in vivo* gene therapy applications. Mutations and variants of SeV have been characterized that allow replication of SeV at a permissive temperature until a temporary shift to a nonpermissive temperature, after which replication is blocked and can no longer be detected.^22,26^ Such control of SeV replication with temperature sensitivity can allow for temporal control of Cas9 and guide RNA expression, which would reduce off-target effects by removing the vector once editing is complete. Mutations that further confer the ability to avoid triggering innate immune responses and concomitant cytopathogenicity would avoid disturbing sensitive cell types such as hematopoietic stem cells or other primary cells.^51,52^ Finally, SeV is amenable to single and multiple deletions of the envelope and/or matrix genes such that the virus can only replicate when these viral factors are supplied *in trans*.^22^ Upon infection of target cells in the absence of these exogenously supplied factors, the virus can produce the factors encoded on its genome but cannot amplify via production of subsequent infectious virus. Many of these innovations have been applied to SeV vectors under development for *ex vivo* and *in vivo* applications, including the commercial SeV-based system for induction of pluripotency to produce pluripotent stem cells^26,32^ (CytoTune-iPS Sendai Reprogramming kit, Invitrogen). Incorporation of these features will be an indispensable next step to develop rSeV-Cas9 as a gene therapy vector.

One advantage of lentiviral and AAV-based platforms are their ability to deliver DNA-based templates for homology-directed repair along with the CRISPR/Cas9 machinery. By virtue of its solely RNA-based lifecycle, SeV cannot similarly encode a donor template in its genome. However, rSeV-Cas9 may still be able to achieve precise editing via cotransfection of a DNA donor. Further, the advent of predictable, targeted “base-editing” via use of a Cas9-cytidine deaminase fusion that effects C-to-T (or G-to-A) substitutions allows precise genome editing in the absence of a DNA donor, and is thus amenable to incorporation in rSeV-Cas9 (ref. 53).

Other paramyxoviruses are also under development as gene therapy and oncolytic vectors. Measles virus and Newcastle disease virus in particular have been closely studied as oncolytic vectors that have shown promise in clinical trials.^45,54,55^ Incorporation of CRISPR/Cas9-mediated gene editing into such vectors may provide an advantage for their oncolytic activity.

In conclusion, this proof-of-principle study opens the door to using SeV, and likely other paramyxoviruses and more generally viruses of *Mononegavirales*, as vectors to deliver Cas9 and its associated guide RNA for efficient gene editing. In addition to the abovementioned advantages, these RNA-based viruses, which exhibit a wide range of relevant primary cell tropisms and lack any risk of genomic integration, can complement existing DNA-based lentiviral and AAV platforms for genome editing–based therapies.

## Materials and Methods

### Cell lines

Flp-In T-REx HEK293 cells (Invitrogen, Waltham, MA), Vero cells (ATCC CCL-81), BSR-T7 cells (BHK-based cell line with stable expression of T7 polymerase),^56^ and Affinofile cells (HEK293-based cell line with inducible overexpression of CD4 and CCR5)^35^ were propagated in Dulbecco’s modified Eagle’s medium (Invitrogen) supplemented with 10% fetal bovine serum (FBS) (Atlanta Biologicals, Flowery Branch, GA) and penicillin/streptomycin at 37 °C. Flp-In T-REx HEK293 cells were additionally maintained in blasticidin and zeocin according to manufacturer protocol, BSR-T7 cells were additionally maintained in 1 mg/ml G418 to maintain the T7 transgene, and Affinofile cells were additionally maintained in 50 µg/ml blasticidin. To generate the mCherry-inducible cells, the mCherry gene was inserted into pcDNA5/FRT/TO and cotransfected with pOG44 (Flp-recombinase) into parental Flp-In T-REx HEK293 cells. Selection with hygromycin (replacing zeocin) and blasticidin according to manufacturer protocol yielded a stable cell line with doxycycline-inducible expression of mCherry.

Whole human blood was obtained from the New York Blood Center. Peripheral blood mononuclear cells were isolated using Ficoll-Paque (GE Healthcare, Boston, MA), and monocytes were further purified using CD14 MicroBeads (Miltenyi Biotec, Bergisch Gladbach, Germany). Monocytes were propagated in Roswell Park Memorial Institute (RPMI) 1640 medium (Invitrogen) supplemented with 10% FBS (Atlanta Biologicals).

### Sendai virus reverse genetics plasmids

The basis for rSeV-Cas9 was our recombinant Sendai virus with an EGFP reporter inserted between the N and P genes via duplication of the N-to-P intergenic region,^33,34^ derived from RGV0 (kind gift of Nancy McQueen), a Fushimi strain SeV with mutations in the F and M genes that allow trypsin-independent growth.^57^ All modifications to the plasmid encoding the T7-driven antigenome were performed using standard and overlapping PCRs with Velocity DNA polymerase (Bioline, Taunton, MA), with subsequent insertion into the construct at unique restriction sites by In-Fusion ligation-independent cloning (Clontech, Mountain View, CA). All cloning was performed with Stbl2 *E. coli* (Invitrogen) with growth at 30 °C. FLAG-tagged codon-optimized *S. pyogenes* Cas9 was amplified from px330^6^ (Addgene, cat #42230, from Feng Zhang) and inserted into rSeV following the EGFP reporter, linked with a P2A ribosomal skipping sequence (ATNFSLLKQAGDVEENPGP). The P2A sequence was preceded by a GSG linker to ensure complete ribosomal skipping.^58^ An additional two nucleotides were added after the stop codon of Cas9 to maintain the rule of six, by which the genome length of paramyxoviruses must be an exact multiple of six to ensure efficient replication.^20^ The Cas9 is flanked by unique NotI and FseI restriction sites to aid in any future modifications. To create the guide RNA and ribozyme cassette, the mCherry-targeting 20 bp sequence was cloned into px330 (see above), and the full chimeric guide sequence (see Supplementary Figure S1) was then PCR-amplified. The hammerhead ribozymes^59,60^ were incorporated via overhangs in the synthesized primers in subsequent PCRs. This cassette was inserted between the P and M genes via duplication of the P-to-M intergenic region, with unique AsiSI and SnaBI restriction sites flanking the cassette to aid in future modifications including changing the guide RNA target sequence (Supplementary Figure S1). The guide RNA target sequences were chosen based on high predicted specificity using the CRISPR design tool (crispr.mit.edu).^46^ The T7-driven helper plasmids encoding SeV-N, SeV-P, and SeV-L were the kind gift of Nancy McQueen.

### Cleavage assay

The efficiency of ribozyme cleavage was determined as previous^61^ with modifications. qRT-PCR primers were designed to flank ribozyme 1 (product A), ribozyme 2 (product B), and within the downstream M gene (product C, representing total RNA) (see Supplementary Figure S4). rSeV-Cas9-mCherry T7-driven antigenome plasmid was transfected into T7-expressing BSR-T7 cells for 2 hours before collection in TRIzol (Invitrogen). Samples were treated with DNase (Invitrogen) at 1 mmol/l MgCl_2_, treated with EDTA, and reverse-transcribed at 1 mmol/l MgCl_2_ with the SuperScript III First-Strand Synthesis System (Invitrogen). qRT-PCR was performed with the SensiFAST SYBR & Fluorescein kit (Bioline), with copy numbers determined by standard curves using the rSeV-Cas9-mCherry antigenome plasmid as template. Percent ribozyme 1 cleavage was determined as 100*((C−A)/C) and normalized to the construct with both ribozymes mutated, and percent ribozyme 2 cleavage was determined as 100*((C−B)/C) and normalized to the construct with ribozyme 2 mutated.

### Viruses and infections

Rescue of replication-competent Sendai virus from transfected plasmid was done as previous^33^ with modifications. BSR-T7 cells in six-well were transfected with 4 µg T7-driven antigenome, 1.44 µg T7-N, 0.77 µg T7-P, 0.07 µg T7-L, and 4 µg codon-optimized T7 polymerase, using Lipofectamine LTX (Invitrogen) according to manufacturer’s recommendations. Virus rescue was monitored by appearance and spread of EGFP fluorescence, and rescued virus was further expanded on BSR-T7 cells. Stocks of clarified virus were stored at −80 °C. Virus titers were determined by titration on Vero cells, with individual infection events detected and counted by EGFP fluorescence at 24 hours postinfection in an Acumen plate reader (TTP Labtech, Melbourn, UK).

For SeV infection of HEK293-based cell lines, 5 × 10^4^ cells were mixed with the virus inoculum immediately prior to plating in poly-L-lysine-coated wells. Media was changed the following day and every 2 days thereafter. For induction of mCherry, 100 ng/ml doxycycline was used. For Affinofile cells, 2 µg/ml ponasterone A and 8 ng/ml doxycycline were used to induce CCR5 and CD4, respectively. For further HIV-1 infection of Affinofile cells, JR-FL HIV-1 was spinoculated onto cells at 2,000 rpm for 2 hours at 37 °C in the presence of 2 µg/ml polybrene (Sigma-Aldrich, St. Louis, MO).

For SeV infection of monocytes, virus stocks were further purified by ultracentrifugation into a discontinuous 20 to 65% sucrose gradient. The interface was collected, titered on Vero cells, and stored at −80 °C until use. 5 × 10^5^ cells in serum-free medium were plated for 30 minutes at 37 °C to allow adherence before infection with virus inoculum via spinoculation at 2,000 rpm for 2 hours at 37 °C. Media was changed to RPMI with 10% FBS following spinoculation and changed every 2 days thereafter. 100 ng/ml granulocyte-macrophage colony-stimulating factor (GM-CSF) (Peprotech, Rocky Hill, NJ) was included in the media following infection to stimulate macrophage differentiation and concomitant upregulation of CCR5.

### Flow cytometry

For CCR5 staining, cells were lifted and blocked in phosphate-buffered saline with 2% FBS. Alexa 647-conjugated rat anti-human CCR5 (cat# 313712, BioLegend, San Diego, CA) was added at 1:100 for 30 minutes at 4 °C before washing and resuspension in 2% paraformaldehyde. For p24 staining (RD1-conjugated mouse anti-p24 clone KC57, cat# 6604667, 1:100 dilution, Beckman Coulter, Brea, CA), cells were fixed and permeabilized using the Cytofix/Cytoperm kit (BD Biosciences, San Jose, CA) before blocking. Flow cytometry was performed on a BD LSR II at the Flow Cytometry Core at the Icahn School of Medicine at Mount Sinai.

### Characterization of mutagenesis

Genomic DNA was extracted using the PureLink Genomic DNA Mini Kit (Invitrogen). Specific genomic loci were amplified using Velocity DNA Polymerase (Bioline) and primers as shown in Supplementary Figure S5. Off-target loci represent the top predicted off-target sites in the CRISPR Design Tool (crispr.mit.edu).^46^ For Sanger sequencing of individual alleles, primers contained appropriate overhangs for insertion between the HindIII and XhoI sites of pcDNA3 via In-Fusion ligation-independent cloning (Clontech). PCR products were gel-extracted (NucleoSpin Gel and PCR Clean-up kit, Clontech), transformed into Stellar competent *E. coli* (Clontech), and selected on ampicillin LB agar. Individual colonies were prepped and sequenced. For deep sequencing, the gel-extracted products were pooled and further prepared for sequencing via paired-end 2 × 300 bp MiSeq (Illumina, San Diego, CA) sequencing by Genewiz (South Plainfield, NJ). Unique sequences were identified and quantified from merged sequenced reads. For each on-target and off-target amplicon reference sequence, 18 bp sequences were selected just beyond 35 bp upstream and downstream from the 20 bp guide RNA target sequence. Unique sequences with exact matches to both of these 18 bp sequences were extracted and collated, with an average of 170,432 reads per amplicon. For each amplicon, sequences with lengths divergent from the reference sequence were identified as having insertions or deletions (indels).
