# Supplementary material for: Sendai virus, an RNA virus with no risk of genomic integration, delivers CRISPR/Cas9 for efficient gene editing
Source: Mol Ther Methods Clin Dev. 2016 Aug 24;3:16057–. doi: 10.1038/mtm.2016.57 (PMC4996130; doi:10.1038/mtm.2016.57)
Supplement: Supplementary Figures [file mtm201657-s1.pdf]

Supplementary Figure 1. Annotated sequence of guide RNA cassette.

P-to-M intergenic region (guide RNA cassette inserted via duplication of this region) (1-118, 325-442)
Gene stop signal (73-83, 397-407)
Intergenic trinucleotide (84-86, 408-410)
Gene start signal (87-96, 411-420)
AsiSI restriction site (unique) (119-126)
Stem for 5′ ribozyme (rbz 1) (127-132, 176-181) - 1st part of stem (GTGGCC in this case) must be the reverse complement of the beginning of the guide RNA targeting sequence. See Fig. 1B for stem structure.
5′ ribozyme (rbz 1) (133-175)
Guide RNA mCherry-targeting sequence (20 bp) (176-195)
TracrRNA (196-271)
Stem for 3′ ribozyme (rbz 2) (272-277, 319-324)
3′ ribozyme (rbz 2) (278-318)
SnaBI restriction site (unique) (320-325)
Mutated to "C" to abolish rbz activity (135 for rbz 1, 299 for rbz 2)

The sequence below is between the stop codon of SeV-P and the start codon of SeV-M.

```
1     ATCCCGGGTG AGGCATCCCA CCATCCTCAG TCACAGAGAG ACCCAATCTA CCATCAGCAT
61    CAGCCAGTAA AGATTAAGAA AAACTTAGGG TGAAAGAAAT TTCACCTAAC ACGGCGCAGC
121   GATCGCGTGG CCCTGATGAG TCCGTGAGGA CGAAACGGTA GGAATTCCTA CCGTCGGCCA
181   CGAGTTCGAG ATCGAGTTTT AGAGCTAGAA ATAGCAAGTT AAAATAAGGC TAGTCCGTTA
241   TCAACTTGAA AAAGTGGCAC CGAGTCGGTG CACGTATCAC CGGAGTCGAC TCCGGTCTGA
301   TGAGTCCGTG AGGACGAAAT ACGTATCCCG GGTGAGGCAT CCCACCATCC TCAGTCACAG
361   AGAGACCCAA TCTACCATCA GCATCAGCCA GTAAAGATTA AGAAAAACTT AGGGTGAAAG
421   AAATTTCACC TAACACGGCG CA
```

For the version containing the hepatitis delta ribozyme (HDV rbz, shown in Figure 2c), the sequence from 272-319 is replaced by the following HDV rbz sequence:
GGCCGGCATGGTCCCAGCCTCCTCGCTGGCGCCGGCTGGGCAACATTCCGAGGGGACCGTCCCCTCGGTAATGGCGAATGGGAC

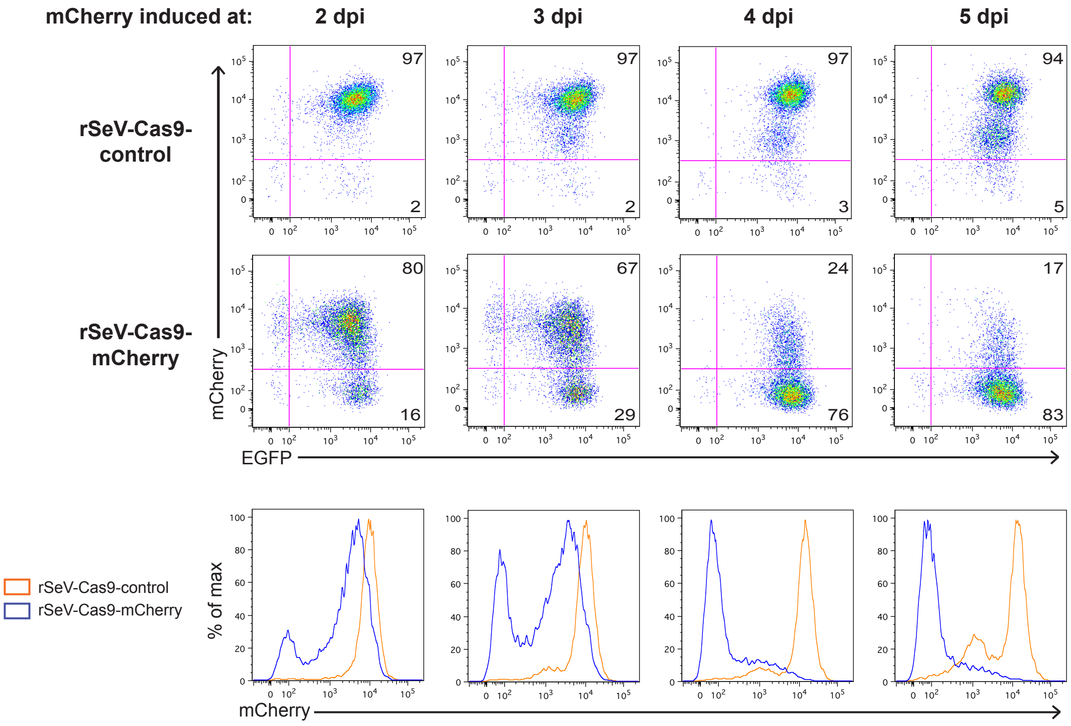

Supplementary Figure 2. Time course of mCherry fluorescence knockout by rSeV-Cas9-mCherry. mCherry-inducible HEK293 cells were infected with rSeV-Cas9-control or rSeV-Cas9-mCherry at MOI 25. mCherry expression was induced with doxycycline at the indicated days post-infection, and cells were collected for flow cytometry the following day. Histograms of mCherry expression (gated on infected GFP+ cells) are shown below as an alternative comparison.

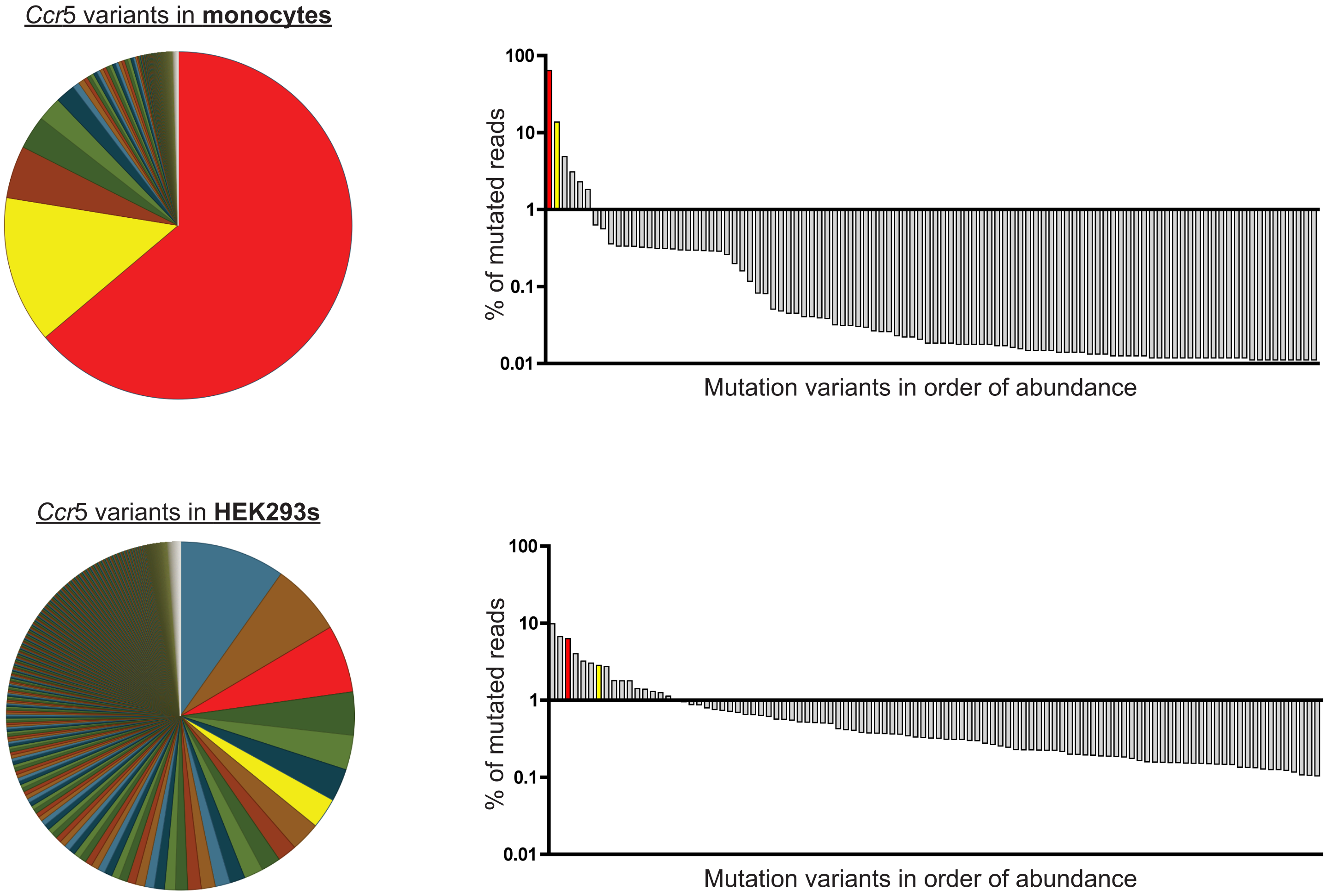

Supplementary Figure 3. Abundance of *ccr5* mutation variants in monocytes and HEK293s. The relative abundance of all mutation variants for *ccr5* are shown in the pie charts, with the 1st and 2nd most abundant variants in monocytes highlighted in red and yellow, respectively. These specific variants are also highlighted in the HEK293 pie chart with the same red and yellow colors. The distributions of variant abundance for the 100 most abundant variants for either monocytes or HEK293s are shown at right, with the two specific variants highlighted above again indicated in red and yellow colors.

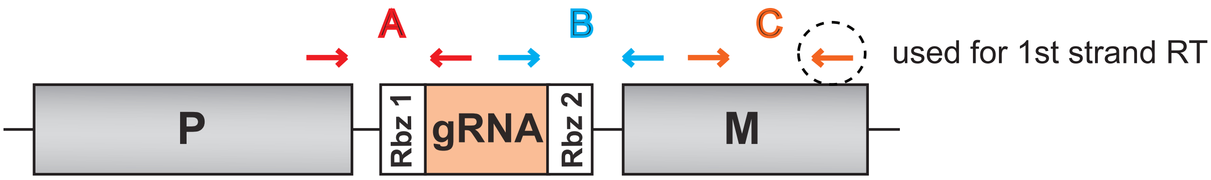

Supplementary Figure 4. Diagram of qRT-PCR primers used for ribozyme cleavage assay.

Supplementary Figure 5. On-target and off-target genomic locations, sequences, and amplification primers.

Sequence	Genomic location	20 bp target + PAM	Amplification primers
mCherry	n/a	GGCCACGAGTTCGAGATCGAGGG	F: GGCGAGGAGGATAACATGG<br>R: CTTCAGCCTCTGCTTGATCTC
ccr5 on-target	chr3, +1<br>46373721	CAGGTTGGACCAAGCTATGCAGG	F: TTGTCATGGTCATCTGCTACTC<br>R: GTGTCACAAGCCCACAGATATT
ccr5 off #1	chr1, -1<br>179505884	...AC....T..........CA.	F: CTCCACTTTCCATAACAGTCTAGG<br>R: GGTCCTTGGAACAGTAGAGATAG
ccr5 off #2	chr3, -1<br>32755718	..A...AC.A...........A.	F: TGTTTGCTGTGAGGCTACTTTG<br>R: TCACTGTCCAATCTGCTTTACC
ccr5 off #3	chr20, -1<br>17216675	A.....TTT...........TA.	F: GCAGAGGCATTATAAACCCAATATG<br>R: CCAGGAGGAACTGGCAAAT
ccr5 off #4	chr2, +1<br>140172166	....A.TC........C...CA.	F: AAGCTCCATCTTCTTCGTTCTT<br>R: AGTAGGAGATGGATTTACAGGTATT
ccr5 off #5	chr12, +1<br>59746621	...T....TT.........TTA.	F: CAGTGACATGAGCACCTGAA<br>R: GCAAGGACATCCTCATCCATAA
efnb2 on-target	chr13, -1<br>106512590	AGAATTCAGCCCTAACCTCTGGG	F: CCTGGACAAGGACTGGTACTAT<br>R: TAGCACAGGGTCCCAAATTC
efnb2 off #1	chr7, -1<br>136299453	.........G.T........TA.	F: GCAGGCTGGTAATTGATCTTTC<br>R: TGATCCACAGTTGGTTGAATCC
efnb2 off #2	chr2, +1<br>36596395	.A.....TT...........AA.	F: CCAGAATGTGTCCTGGGTTTAG<br>R: GTGTCAGAGCGAGACTTTGT
efnb2 off #3	chr7, +1<br>98992870	.C.T......T............	F: GGAGTATCTTCAGCTGTGAGAAG<br>R: CTGTTACACGTTCCTTGCTACT
efnb2 off #4	chr14, +1<br>98564083	.T..A...........A....A.	F: CTGATTGAGTGGGTCATCAGAA<br>R: GCTACGTGCTGGTGCTAAA
efnb2 off #5	chr3, -1<br>6909191	.A..G.TT............CA.	F: GTCCAGGAAAGAAAGTTGCATAAG<br>R: GCTGTCTGCTGGAAAGATAGT
